# Perceptions of and barriers to vaccinating daughters against Human Papillomavirus (HPV) among mothers in Hong Kong

**DOI:** 10.1186/1472-6874-14-73

**Published:** 2014-06-02

**Authors:** Judy Yuen-man Siu

**Affiliations:** 1 David C. Lam Institute for East–West Studies (Environment, Health, and Sustainability working group), Hong Kong Baptist University, Kowloon, Hong Kong

**Keywords:** HPV vaccination, Perceptions, Barriers, Mothers, Daughters, Hong Kong

## Abstract

**Background:**

Significant others are noted to be remarkable influences in modelling children’s and young people’s health perceptions and their adoption of health behaviour. The vaccinations which a child receives are shown to be significantly influenced by his or her parents. However, there is a paucity of Chinese-based studies. When discussing the Human Papillomavirus (HPV) vaccine, very few studies examine the perceptions of Chinese parents regarding the vaccine as a preventive health measure, and even fewer examine how these perceptions of the vaccine and sexual values influence their motivations in encouraging their children to be vaccinated. In view of the literature gap, this article investigates the perceptions of Hong Kong mothers in regard to vaccinating their daughters against HPV in Hong Kong.

**Methods:**

A qualitative research approach with individual semi-structured interviews was conducted with 35 mothers aged 30 to 60 years old with daughter(s) between 9 and 17 years old.

**Results:**

Six connected themes emerged. The participants commonly perceived the HPV vaccination as being unnecessary for their daughters in view of their young age. They worried that it would encourage their daughters to engage in premarital sex, and perceived the vaccination to be potentially harmful to health. Also, their low perceived risk of HPV in addition to the lack of reassurance from their health care providers failed to convince the participants that the vaccination was important for their daughters’ health. Finally, the participants found the vaccine to be expensive and perceived it to have little protection value in comparison to other optional vaccines.

**Conclusion:**

The sampled mothers did not have a positive perception of the HPV vaccine. The cultural association between receiving the vaccination and premarital sex was prevalent. Bounded by their cultural values, the participants also had many misconceptions regarding the vaccine and the transmission of HPV, which discouraged them from having their daughters vaccinated. Furthermore, a lack of support from health care providers and the government health authorities concerning HPV vaccination failed to provide confidence and reassurance to mothers, and conveyed a meaning to these mothers that HPV vaccine is relatively unimportant.

## Background

Cervical cancer is the second most common cancer in women worldwide [1]. In Hong Kong, it was the ninth most common cancer among women in 2011, and the eighth most common cause of female cancer deaths in the same year in Hong Kong [2]. Cervical cancer accounted for 3% of all new cancer cases in women and 2.8% of female cancer registered deaths in Hong Kong [2]. Statistics show that the morbidity of cervical cancer was among the highest in the age group of 20 to 44 [2].

Cervical cancer is caused by the genital infection Human Papillomavirus (HPV) [1], and HPV-16 and 18 are the high-risk strains which are responsible for over 70% of cervical cancer cases [3,4]. Besides cervical cancer, HPV can also lead to cancers of the vulva, vagina, penis, anus and oropharynx, as well as genital warts [5].

The HPV vaccination has been clinically recognized as an effective preventive measure in decreasing the morbidity of cervical cancer [6-8]. The vaccine is most effective when given to women who have not yet become sexually active and younger women, especially below the age of 18 [9]. It is therefore highly recommended for adolescent girls and young adult women [10]. Many countries have implemented HPV vaccination programmes to reduce the burden of HPV-associated diseases [11,12].

In Hong Kong, HPV vaccines Gardasil® and Cervarix® have been available since 2006 [13]. The vaccine is recommended for women aged 9 to 45 years old [14]. The vaccine is voluntary, and is not included in the compulsory Hong Kong Childhood Immunization Programme by the Department of Health [15]. Those who receive the HPV vaccination thus have to turn to private-practice clinics on a self-pay basis. A three-dose course usually costs between HK$2,500 (equivalent to US$321) and HK$4,000 (US$514). Unlike other optional vaccines such as the seasonal influenza vaccine and pneumococcal vaccine, no subsidies were provided by the government for receiving the HPV vaccine at the time of this study. However, since 2011, under the Youth HPV Prevention Programme supported by The Society of Physicians of Hong Kong and The Family Planning Association of Hong Kong, primary and secondary school students now enjoy a discounted rate of $2,000 (US$256) for the whole vaccination course [14]. Students in tertiary education can also receive a discounted rate at university health centres, though prices vary. In 2008, 768 women received the HPV vaccine from The Family Planning Association of Hong Kong [16], and around 5% of the target population was vaccinated by 2011 [17].

### Significance

Studies suggest that 75% of sexually active people will be infected with HPV at some point during their lives [18]. However, acceptance of the HPV vaccination is still lacking, both in Western communities [19,20] and non-Western communities like Hong Kong [16,17]. Hence, understanding women’s perceptions about HPV and the HPV vaccine is important to encourage more comprehensive vaccination coverage.

Significant others have notable influence in shaping the health perceptions of children and teenagers [21], who learn from their parents about health matters [22]. Moreover, decisions about children’s treatment and preventive health care are largely made by their parents [23]. In an earlier study examining perceptions of the HPV vaccination among female university students in Hong Kong [24], parents – and in particular mothers – are noted to be decisive in preventing young women from receiving the vaccination. Although parents have been shown to play a significant role in children’s health behaviour, there have been few studies about the perceptions of Chinese parents toward the HPV vaccination, and even fewer concerning how these perceptions influence their decision on whether to have their children vaccinated. The few past studies on this subject [25-27] were conducted in non-Asian countries, and there is a paucity of Chinese-based research.

Many young adults are ignorant about the HPV vaccination as a preventive health measure [28], largely influenced by their parents’ lack of awareness [29,30]. As a result, educating parents about the benefits of preventive health measures is very important to children’s health [31]. This study investigates how mothers in Hong Kong perceive the idea of vaccinating their daughters for HPV, to enable an in-depth understanding of their concerns and thus better inform future efforts to enhance Hong Kong women’s awareness of the HPV vaccination.

## Methods

### Data collection

A qualitative approach was adopted using individual semi-structured interviews. Thirty-five mothers were recruited through purposive sampling [32] in a health talk about breast cancer prevention organized by a women’s social service agency in Hong Kong. With the approval of the organizer, each attendee of the talk was given an information sheet and a short questionnaire asking about the sampling criteria (see the below paragraph for the inclusion criteria) and their contact methods if they consented to participate in the study. Those who fulfilled the sampling criteria and who left their contact methods were contacted individually, and 35 participants were sampled between March and April 2013.

Purposive sampling of the participants was conducted according to the following inclusion criteria: (a) women aged 30 to 60 years at the time of study, who (b) had at least one daughter aged 9 to 17 years old at the time of the study, (c) had not yet taken their daughter(s) to receive the HPV vaccine, (d) had no professional medical and/or health science training, (e) had the ability to understand and speak Cantonese, and (f) were Hong Kong Chinese by ethnicity. Because the HPV vaccine is mainly targeted at women who are not yet sexually active, and because mothers are noted to be among the most important significant others in influencing the health perceptions and behaviour of their children [33], mothers with the aforementioned characteristics were purposively sampled to examine their perceptions of the vaccine. To investigate the perceptions of the general populace, those working in medical and health care professions were excluded from the sampling.

Prior to the interviews, the 35 participants were informed about the purpose and nature of the study with a participant information sheet written in their mother tongue. Written consent was obtained from each of the participants. They could seek clarifications before the interview, and were assured of their rights and freedom to withdraw from the study at any time. All interviews were conducted in a private room in the women’s social service centre, and were audio-recorded with the participants’ consent. Cantonese Chinese was used as the medium of conversation, and was the mother tongue of all the participants. To protect their privacy and confidentiality, no names or identities were mentioned throughout the interviews.

The interviews were conducted between March and June 2013, and lasted between 1.75 and 2 hours. I conducted all the interviews to ensure interview consistency and quality. I developed an interview question guide (see “Additional file 1”) for use in the interviews to direct the discussion and ensure that the interview stayed focused on the topics and followed an appropriate direction. Participants’ demographic data, including their age, education level, occupation, marital/relationship status, number and age of their daughters, and doctor-seeking habit, were obtained at the end of the interviews. To compensate for their time, each participant was given a HK$100 supermarket cash coupon as an acknowledgement upon completion of the interviews.

### Ethics considerations

The study obtained research ethics approval from the Committee on the Use of Human and Animal Subjects in Teaching and Research at Hong Kong Baptist University. All data and field notes were stored in locked files and treated with strict confidentiality. Only I had access to the data and field notes. Each participant was represented as a code in the data and interview transcripts to further protect the privacy and confidentiality of the participants. The audio-digital records of the interviews were destroyed immediately after the interviews had been transcribed and checked for accuracy.

### Data analysis

Quick data analysis was conducted during the interviews to ensure what was known and what needed to be explored further [34]. This involved constant checking of the questions in the interview question guide and a quick assessment during the interviews to determine if further probing was needed. Interviews were transcribed verbatim within seven days of each interview, and coding and analysis were performed to determine data saturation. Interview transcriptions were segmented into meaning units, and thereafter collapsed into categories and eventually themes through the process of abstraction and constant comparison. Repetitive themes were highlighted. Coding schemes were developed [35] according to the inductive coding process by allowing the identification of behavioural and thinking patterns [32]. A code book was kept to record special data and to transform the data from interviews into categories to identify major themes [32]. New thematic codes emerging from the data were added to the coding list, and the codes were grounded in the data.

### Rigour of the study

The rigour of the study followed the criteria suggested by Lincoln and Guba [36]. Validity-checking with the participants was performed to establish credibility. The participants were asked to check the transcribed interviews to ensure that the transcriptions did not distort their meanings [34]. Interview quotations from the participants were included in the coding table so that their ideas were clearly represented. This also ensured cross-checking between the interview quotations, themes, and categories throughout the analysis. Neutrality in the analysis was established at the same time, since having interview quotations in the coding table ensured that the findings were grounded in the data. As I was the only researcher involved in data collection and analysis, coding and recoding of the transcripts were performed to ensure data reliability, and that the categories and themes were free of ambiguity, bias, overlap and lack of clarity. Recoding was performed a month after the first coding to ensure the accuracy of the categories and themes.

## Results

### The participants

All 35 participants were members of a social service centre for women, and ranged between 30 and 60 years old. Each had at least one daughter between 9 and 17 years old living with them. Neither they nor their daughters had ever received the HPV vaccine. Twenty-four participants were married, nine of them were divorced and two of them were widowed.

Twenty-one of the participants were employed full-time in different sectors, including administrative and executive functions, sales and retail, food and catering, education, social service, civil service, transportation and logistics. Five participants were employed part-time in sales or catering. Nine of the participants were homemakers.

All the participants had received some level of formal education: 11 participants had completed university, two had attained post-secondary school education, 20 had reached form 5 in secondary school and two had reached form 3.

It was not common for participants to have a regular family doctor. Only eight participants claimed to have one, while 17 participants would go to the same clinic (but not necessarily the same doctor) when sick. The remaining ten participants doctor-shopped whenever they needed medical consultation.

### Themes: What did the sampled mothers think about vaccinating their daughters against HPV?

Six themes were identified in relation to the participants’ perceptions of vaccinating their daughters. Overall, these perceptions intertwined to discourage the mothers from having their daughters receive the HPV vaccine.

#### HPV vaccination unnecessary for their daughters

Because of the young ages of their daughters, all the participants perceived the HPV vaccination as being unnecessary. The vaccine was widely perceived to be mainly for women who were about to become sexually active, and therefore these mothers did not think that their daughters needed to be vaccinated. One participant said:

My daughter is still very young. She is just 15 years old, and I assume she will only get married when she is in her late 20s or early 30s. The cervical cancer vaccine is for preventing cervical cancer, so it is for those who are about to start their sexual life. I have never thought about taking my daughter to be vaccinated. It is too early for her [P27].

The effective duration of the vaccine remained unknown to the participants, and therefore they believed that their daughters could wait till just before marriage to receive the vaccination. This participant indicated a common perception:

My daughter is still very young, so I think that there is no need for her to be vaccinated at the moment. It is too early for her; she is just 11 years old now! Also, I am not sure how long the vaccine efficacy can last. If the efficacy can just last for five years, then it is of no use to her to be vaccinated now. She will just turn 16 years old at that time and there are still many years ahead for her to get married. There will not be any point if she is vaccinated now; the vaccine will just be wasted [P6].

The way in which participants perceived the HPV vaccine also influenced their motivations. All the participants referred to the vaccine as the “cervical cancer vaccine”. Because cervical cancer is a disease that affects adult women who are sexually active, this naming reinforced the participants’ perceptions that the HPV vaccine was for adult women rather than teenage girls:

As far as I know, only adult women get cervical cancer, so I think the cervical cancer vaccine is mainly for adult women. I have never heard of young girls getting cervical cancer. If young girls do not get cervical cancer, then what is the point of taking my daughter to be vaccinated? She is just 13 years old [P5].

#### HPV vaccine will encourage premarital sexual behaviour

Another common perception among the sampled mothers was that the vaccination had the potential to encourage premarital sexual behaviour. Almost all the participants worried that vaccinating their daughters could convey the message that they were allowed and approved to begin their sexual lives. This worry was particularly prevalent among participants with daughters in their teenage years. One participant expressed her concern:

I do not think that it is good to vaccinate my daughter at the moment. The cervical cancer vaccine is mainly for women who are about to embark on their sexual life. She has just entered puberty, and taking her to have the vaccine may make her misunderstand that premarital sex is acceptable. My daughter is studying in a co-educational school; therefore she will see her male classmates every day, and she may be dating sooner or later. If I take my daughter to be vaccinated, I am afraid that this will send the wrong message to her that it is now safe to have premarital sex [P14].

In many cases, the participants perceived that making their daughters fear contracting a disease could be effective in preventing them from having premarital sex. This reinforced the participants’ idea that their daughters should not be vaccinated, so they would be frightened of the health risks. As this participant explained:

Taking my daughter to be vaccinated may make her perceive wrongly that she will not have any health consequences when considering premarital sex. Therefore I am very reluctant to take my daughter to be vaccinated. Not taking my daughter to be vaccinated will make her think more about the health consequences, such as getting cancer from having premarital sex. I think that this is more effective in educating my daughter about the bad consequences of having premarital sex. If she is vaccinated, then disease and cancer will no longer scare her away from thinking about having premarital sex [P19].

#### HPV vaccination as potentially harmful to health

More than half of the participants perceived the HPV vaccine as being harmful to one’s health. They thought that most vaccines are either manufactured from a virus or from artificial chemicals; therefore, they felt reluctant about taking their daughters to receive an additional but optional vaccine:

Vaccines are made from viruses. Therefore, I believe it is not good to have too many vaccinations. After all, a vaccine is foreign material, and this material can make your body weak. You may get sick after the vaccination, and that’s why we often feel uncomfortable and have a fever after vaccination, because we get sick from the vaccine. If the vaccine is compulsory, then I have no choice. However, the cervical cancer vaccine is optional, so I do not want my daughter to get sick from the vaccine [P24].

The HPV vaccine was a new vaccine to all the participants. They expressed doubt and hesitation about its safety. Most of them did not know much about this vaccine, except for the side-effects that had been reported in the newspapers. As this participant recalled:

It seems that the cervical cancer vaccine is quite new. New things are always not good and always need adjustment and modification. I do not want my daughter to be a “white mouse”. Also, I remember that there have been some side-effects reported in the newspapers when this vaccine was just out on the market. I am not very confident about the safety of this vaccine at the moment, and so do not want my daughter to take the risk. Maybe after some more years, when the safety of this vaccine has been proved, then I may consider taking my daughter to be vaccinated, but definitely not at the moment [P18].

#### Low perceived risk of the dangers of HPV

Most of the participants had little understanding or knowledge about HPV and its potential danger to women’s health. Many of them had never heard of HPV, and were not aware of the close relationship between HPV and cervical cancer. One participant said:

I do not know what HPV is, though I know about cervical cancer. Cervical cancer is caused by sex, so I do not think that receiving the vaccination can help you to prevent cervical cancer. I think that not having sex should be the best way to prevent cervical cancer rather than receiving the vaccination [P2].

The low perceived risk about the dangers of HPV reduced the participants’ incentive to have their daughters receive the vaccination, and their daughters’ young ages lowered the perceived risk even further. As this participant indicated:

Cervical cancer is caused by sex. My daughter is just 12 years old, and still has a long time before she gets married. Therefore I do not think that she will have any possibility of getting cervical cancer in these coming few years. I think that the vaccine is more useful to those women who will get married soon. In my daughter’s case, I cannot see what the vaccine can do for her, since she does not have any risk of getting cervical cancer [P20].

The participants’ low perceived risk about the dangers of HPV was reflected in their own lack of regular cervical screening for themselves. Many of them did not think it necessary, since they believed the likelihood of suffering from cervical cancer was low. Such lack of awareness about the importance of this preventive health measure also affected their opinion about the HPV vaccine for their daughters. This participant indicated popular thinking:

I know about pap smears, but I do not think that it is necessary for me. I only have my husband [as a sexual partner] and no other men. Only promiscuous women need to have pap smears, because they can more easily get cervical cancer. I believe that if a woman is moral and faithful, she does not need to worry about cervical cancer and having pap smears. It is just the same case for my daughters. If they are moral and faithful, then they do not need to worry about cervical cancer. Only those who are promiscuous need to be vaccinated. I trust that my daughters will be morally behaved and be good women when they grow up [P29].

#### Lack of reassurance by health care providers

Health care providers’ lack of reassurance about the HPV vaccine is a significant barrier to the participants considering whether to vaccinate their daughters. Almost none of the participants’ health care providers encouraged the vaccination proactively. This lack of promotion often reinforced the perceptions of the participants that the HPV vaccine was unimportant and unnecessary:

Every time I accompany my daughter to see our family doctor, he does not mention anything about the cervical cancer vaccine. However, he encourages us to receive the influenza vaccination and pneumonia vaccination [pneumococcal vaccine]. I trust our family doctor, and he will not ask us to have any unnecessary vaccination. It seems to me that the influenza and pneumonia vaccinations are more necessary to health than cervical cancer vaccination. That’s why I have never thought about taking my daughter to receive this vaccination [P11].

Difficulty in obtaining information from health care providers is another significant barrier. In some cases, participants had asked about the vaccination for their daughters, but the non-enthusiastic responses from their health care providers further discouraged them:

I once asked our family doctor about the cervical cancer vaccine. However, the doctor seemed to not know much about the vaccine. He just asked us to read the pamphlet outside. Perhaps my daughter was quite sick last time, so he focused more on treating her that time. After all, we see doctors only when we are sick. As a mother, when my daughter is sick, I will just focus on hoping that the doctor can treat my daughter, rather than thinking about a non-urgent vaccination [P25].

#### Vaccination as expensive in relation to low protective value

These mothers commonly perceived the vaccination to be expensive. However, the expense was not a standalone factor in the participants’ decision not to have their daughters vaccinated; rather the financial issue had to be combined with other factors. Because the vaccine is not included in the government’s compulsory Childhood Immunization Programme, it conveys the impression that the HPV vaccination is unnecessary and unimportant for their daughters’ health. As this participant indicated:

The cervical cancer vaccine seems to be not very necessary. Those necessary vaccines should be compulsory and included in the government’s child vaccination scheme. There are so many new vaccines nowadays, and they all cost money. If the vaccine is really necessary, I believe that the government’s child vaccination scheme will include it without charge. Those vaccines that need to be paid for should be unnecessary [P9].

More than half the participants perceived the protection value of the HPV vaccine to be lower than other self-pay optional vaccines such as the seasonal influenza vaccine and pneumococcal vaccine. In their minds, influenza and pneumonia were more acute and more easily transmitted, and had the potential to impose an immediate life-threatening impact on their daughters’ health. In comparison to the more distant impacts caused by HPV, participants’ risk perception of HPV-related diseases was thus lower than these more acute diseases. This perception failed to motivate the participants to take their daughters for the HPV vaccination. As this participant shared:

Unlike other vaccines that need to be paid for, the protection value of the cervical cancer vaccine seems to be the least. I am more motivated to take my daughter to receive the pneumonia [pneumococcal] vaccine, because pneumonia can kill and it can be easily transmitted through air. Even if it does not kill, still it can make my daughter be absent from school. Therefore it is more important to have the pneumonia vaccine. Cervical cancer is not that easily transmitted, unless you have sex. My daughter is still young, and still has a long time before getting married, so I do not think that she can get cervical cancer in the near future. I do not think that the protective value of the cervical cancer vaccine for my daughter is high enough to motivate me to take her to be vaccinated [P23].

## Discussion

In contrast to other international examples which often show parents’ support for the HPV vaccine [37], in Hong Kong a number of social and cultural perceptions intertwine to prevent the participants of this study from encouraging their daughters to receive the vaccine. They commonly perceived the HPV vaccination as unnecessary in view of their daughters’ young ages. They were also concerned that it could encourage premarital sexual behaviour and thought the vaccination was potentially harmful to health. Their low perceived risk of the dangers of HPV in addition to the lack of reassurance by their health care providers failed to convince the participants that the vaccination was important to protect their daughters’ health. Finally, the participants perceived the vaccine to be expensive, with little protection value in comparison to other optional self-pay vaccines. The socioeconomic status of the participants – including education level and occupation – made little difference to their impressions. However, their cultural values about women and sexuality were prominent in shaping their perceptions.

Because of their daughters’ young ages, the participants commonly perceived the HPV vaccination to be unnecessary. Cervical cancer has been represented as a sex-induced cancer in Hong Kong [38], and is often portrayed as a disease that exclusively afflicts sexually active women. As noted by Sontag [39], many diseases have attached a negative connotation, and cervical cancer is no exception. Suffering from cervical cancer often symbolizes a woman’s promiscuity, suggesting multiple sexual partners and a sex life started young [38]. These connotations are closely associated with sexually active adult women, and therefore the stereotype persuaded the participants against the belief that the HPV vaccine was important to their daughters’ health at this early stage.

Language use can powerfully shape people’s cultural beliefs and perceptions [40]. Participants often referred to the HPV vaccine as the “cervical cancer vaccine” in interviews, and this designation influenced how the participants perceived it. The name conveys an impression that the vaccine is specifically for the prevention of cervical cancer. Because cervical cancer primarily afflicts sexually active women, it strengthened their perception of the interlocking relationship between sexual activity, HPV and cervical cancer, thereby reinforcing the belief that their daughters, who were still young, were not at risk. This served to discourage the participants from taking their daughters to receive the vaccination. The “cervical cancer vaccine” as understood by the participants, was only needed by women who had begun their sexual lives.

The belief that their daughters’ had a low risk of contracting HPV impacted the participants’ perceived need for the vaccine. The risk perception correlated with sexual experience, and sexual experience was deemed to be positively related to age. Because their daughters were young, the participants assumed that they were sexually inactive, and thus had no need for the HPV vaccination [41,42]. However, this belief could put their daughters at risk, since adolescents can of course be sexually active [43]. The participants’ assumption of their daughters’ abstinence is therefore unfavourable in the prevention of HPV-associated diseases in young populations.

For the sampled mothers, the HPV vaccination had a symbolic meaning. The vaccine was commonly perceived to be for sexually active (or soon-to-be sexually active) women; hence, receiving the vaccination was deemed to be almost a rite of passage for their daughters, conveying approval to begin their sexual lives. Concerned about the possibility of transmitting such a message to their daughters, this proved to be a prominent barrier to the participants against the HPV vaccination. Indeed, unfavourable perceptions about vaccinating women who are not sexually active are not uncommon in Hong Kong [42], and the participants’ worries about the possibility of their daughters engaging in premarital sex after being vaccinated were notable. Patriarchal sexual values prevail in Hong Kong [44], and virginity is an important cultural ideal for unmarried women in Chinese communities. Because the HPV vaccine possesses a symbolic meaning that violates this Chinese cultural value, it is unsurprising that the participants were not supportive of the vaccination for their daughters. Yet due to this value, both participants and their daughters were deprived of the right to choose better health protection for themselves, resulting in health inequality [45] that is caused by this gender ideology. Young women have been shown to be heavily influenced and regulated by their parents regarding sexual perceptions and sexuality [46,47]. The participants’ beliefs about the HPV vaccine would undoubtedly influence their daughters, instilling in them similar perceptions [24].

In patriarchal societies, women are under different forms of social control [48]. Medicine is a major institution of social control [49], and the notion of women’s diseases has been a common form of control over women’s bodies and behaviour [50,51]. Women are socially constructed as a “diseased body” [52], and they are to be blamed if they fall sick. These social constructions control women’s behaviour [52], and women are perceived as the ones who should bear the primary responsibility for undertaking preventive health measures [53]. During the fieldwork for this study, little mention was made of the man’s role in preventing or transmitting HPV. Most promotion about the vaccination is targeted at women rather than men. In the interviews, none of the participants were aware of the man’s role in HPV transmission, and none of them were aware that men also need to be vaccinated to prevent further transmission. Instead, they commonly believed that women bore the main responsibility in preventing HPV by disciplining themselves, behaving morally and maintaining abstinence.

Cervical cancer was often perceived by the participants as closely associated with a woman’s promiscuity and immorality. These patriarchal values controlled the participants’ health values and health behaviour, not only leading them to believe that they did not need regular cervical screenings, but also leading to their lack of awareness about men’s role in HPV transmission and the importance of having their daughters vaccinated. Such patriarchal ideology was prevalent among the participants of all education levels. This represents a definite risk for women’s health and disease prevention. These mothers transmitted and executed these cultural beliefs as social control over their daughters. They perceived that the health consequences might scare their daughters away from premarital sexual activity, and as a result, the participants lacked incentive to encourage their daughters to be vaccinated.

Consistent with previous studies showing that parents are concerned regarding the HPV vaccine’s effectiveness, safety and side-effects [12,54], the participants also worried that the vaccination might not be safe for their daughters, in view of the vaccine’s short history. New vaccines are shown to cause worry, hesitation and suspicion [24]. The cultural stereotype of vaccines as “a virus and a foreign intruder to the body” was prominent among the participants. In conjunction with the reinforcement by media reports about the vaccine’s side-effects, the participants’ concerns further persuaded them to reject the HPV vaccine.

Doctor recommendations are documented to be a significant incentive for women to receive the HPV vaccination [41,55]. Consistent with international literature, insufficient information and support from primary care doctors was another profound barrier for the participants [56,57]. Although one of the key roles of primary care doctors is the provision of disease prevention education and health care management advice [58], many of the participants were not aware of this role. The participants still possessed a traditional understanding of the role of doctors as treatment providers, and they consulted a doctor only for treatment when they fell sick. Obviously there was a disconnection between participants’ understandings and the more recent literature on the roles of primary care doctors. Therefore, more patient education regarding the different roles of primary care doctors and enhancing primary care doctors’ enthusiasm to provide detailed information about disease prevention and promote preventive health will help to motivate women’s vaccination behaviour.

The focus of vaccination promotion among primary care providers also supports the supposed triviality of the HPV vaccination. In addition to the HPV vaccine, the seasonal influenza vaccine and pneumococcal vaccine are also optional self-pay vaccines in Hong Kong. Doctors’ emphasis on promoting those vaccines over the HPV vaccine further reinforced the participants’ belief that the HPV vaccine is less vital than other optional vaccines. Primary care providers are an important agent in influencing people’s perceptions of a vaccine [59]. Their emphasis on vaccination promotion is closely related to their knowledge about a vaccine [60]. Therefore, enhancing doctors’ knowledge about the HPV vaccine and its importance to women’s health may motivate them to encourage HPV vaccination, providing reassurance to parents and encouraging them to have their daughters vaccinated.

The vaccination subsidy policy of the government health authorities also reinforced the participants’ perceptions about the relative insignificance of HPV vaccination. The government provides subsidies for the seasonal influenza and pneumococcal vaccines, but not the HPV vaccine. Superficially, the subsidies served as a financial incentive to the participants. However, the subsidies also convey a symbolic meaning, influencing perceptions about the importance of a vaccine. The lack of government subsidies for the HPV vaccine therefore reinforced the participants’ perception that this vaccine is unnecessary for their daughters’ health. Indeed, the government’s vaccination policy has a prominent role in influencing people’s vaccination behaviour [61], and the lack of government’s support for the HPV vaccination in Hong Kong thus negatively impacts parents’ incentive to have their daughters vaccinated.

The high cost of the HPV vaccine was another notable barrier for the participants. To some extent, this shows consistency with previous studies [62,63]. However, the expense barely served as a standalone barrier to the participants, as being “expensive” is a relative concept that needs to be weighed against function and worth. The value of the vaccine is not determined by price, but instead by its potential to provide useful protection. Therefore, discounts and lower vaccination costs may only produce limited results in motivating parents to have their daughters vaccinated.

Parents are the role models for their children, and they play a significant role in constructing and modelling their children’s health perceptions and preventive health behaviour [22]. The perceptions of the participants would presumably influence the perceptions and motivations of their daughters to receive the HPV vaccine. As noted, the participants themselves did not receive HPV vaccination, nor were they very aware of the benefits of regular cervical screening as a preventive health measure against cervical cancer. Their misconceptions about the low risk of HPV influenced how they communicated the risk of HPV to their daughters, and transmitted their lack of vaccination incentive. Echoing past literature, the daughters’ HPV vaccination uptake was closely correlated with their mothers’ cervical screening habit. If mothers do not engage in such preventive health measures, their daughters are less likely to be vaccinated [64]. Earlier studies show that young women in Hong Kong are demotivated from receiving HPV vaccination, partly due to their parents’ opposition, and partly due to the perceptions and values that have been passed on from their parents [24]. To effectively promote this preventive health behaviour, parents – and in particular mothers – should be educated about the risk of HPV and reassured about the positive value of the HPV vaccine. If significant others are not convinced about the importance of the vaccine, children can hardly be motivated to receive it. In the future, HPV vaccination promotion should aim at educating parents with more positive values in order to enable this preventive health screening to reach a wider adolescent population in Hong Kong. This may be able to overcome the influence of patriarchal values that prevail in Hong Kong.

### Limitations

The sampled participants comprised only mothers aged 30 to 60 years at one centre of a women’s social service agency. Other mothers were not included. Future studies sampling mothers from more field sites can add more credibility to understanding the perceptions of mothers regarding the HPV vaccine in Hong Kong. Moreover, because many participants experienced a lack of support from their primary care providers, future study about primary care doctors’ knowledge and understanding of HPV, as well as their health promoting behaviour regarding HPV prevention, can allow for a deeper understanding of the experience and behaviour of the participants.

## Conclusion

Promoting HPV vaccination among adolescent girls and young women is an important public health measure in the prevention of cervical cancer and HPV-associated diseases. Yet adolescent girls do not have full independence in making decisions about their preventive health, and because they are still under the socialization of their parents, their parents’ perceptions and beliefs have a decisive role in constructing these adolescents’ health perceptions and shaping their preventive health behaviour. This article examines a number of perceptions among the sampled mothers about the HPV vaccine. These perceptions, combined together, served to prevent the participants from encouraging their daughters to receive the HPV vaccine. The participants did not have a positive perception of the HPV vaccine. The cultural association between receiving the vaccination and premarital sex was prevalent. Bounded by their cultural values, they also had many misconceptions regarding the vaccine and the transmission of HPV, which discouraged them from having their daughters vaccinated. Furthermore, a lack of support from health care providers and the government health authorities concerning HPV vaccination failed to provide confidence and reassurance to mothers, and conveyed a meaning to these mothers that HPV vaccine is relatively unimportant. The data show that the sampled mothers are in need of education about the risk of HPV and further assurance about the vaccine, so that they can transmit a more positive message to their daughters in regard to undertaking this preventive health measure.

## Competing interests

The author declares that she have no competing interests.

## Pre-publication history

The pre-publication history for this paper can be accessed here:

http://www.biomedcentral.com/1472-6874/14/73/prepub

## Supplementary Material

Additional file 1 Interview question guide of the study. Click here for file
