# Supplementary material for: Perceptions of and barriers to vaccinating daughters against Human Papillomavirus (HPV) among mothers in Hong Kong
Source: BMC Womens Health. 2014 Jun 2;14:73. doi: 10.1186/1472-6874-14-73 (PMC4049476; doi:10.1186/1472-6874-14-73)
Supplement: Additional file 1 — Interview question guide of the study. [file 1472-6874-14-73-S1.pdf]

Table 1. Interview question guide of the study

A. **About perception (substitute "HPV" and "HPV vaccines" with the terms that the informants use in the interviews)**
   1. Have you ever heard about HPV?
   2. How do you think about the transmission route of HPV?
   3. How do you think about the danger of HPV?
   4. Have you ever heard about HPV vaccine?
      - If yes, from what sources you have heard about the vaccine?
   5. How do you think about the use of HPV vaccine?
   6. Have you ever received HPV vaccination?
      - Why?
   7. Have you ever received women's health check-up, such as cervical screening?
      - Why?
      - If yes, how often?

B. **About vaccinating their daughter(s) (substitute "HPV" and "HPV vaccines" with the terms that the informants use in the interviews)**
   8. To you, who should receive the HPV vaccine?
   9. Do you think all women should receive the HPV vaccine?
      - Why?
   10. Have you ever thought of bringing your daughter(s) to receive the vaccination?
       - Why?
   11. How do you think about the needs of your daughter(s) in getting vaccinated?
   12. What are the reasons for you not bringing your daughter(s) to get vaccinated?
   13. To you, when is the suitable time / age for your daughter(s) to receive HPV vaccination?
   14. Has any person (such as health care providers) ever discussed with you about vaccinating your daughter(s) against HPV?
       - How do you think about these suggestions?
   15. How do you think about the danger of HPV to your daughter(s)?
   16. How do you think about the use of HPV vaccine to your daughter(s)?

17. How do you think about the side effects / potential harm / disadvantages on your daughter(s) if she / they receive(s) HPV vaccination?
18. How do you think about the advantages on your daughter(s) if she / they receive(s) the vaccination?

C. Demographic background of sampled mothers

19. Age
20. Marital / relationship status
21. Education level
22. Occupation
23. Religious belief
24. Having a family doctor or not
25. Number of their daughter(s)
26. Age of their daughter(s)
